# Platelets Independently Recruit into Asthmatic Lungs and Models of Allergic Inflammation via CCR3

**DOI:** 10.1165/rcmb.2020-0425OC

**Published:** 2021-05

**Authors:** Sajeel A. Shah, Varsha Kanabar, Yanira Riffo-Vasquez, Zainab Mohamed, Simon J. Cleary, Christopher Corrigan, Alan L. James, John G. Elliot, Janis K. Shute, Clive P. Page, Simon C. Pitchford

**Affiliations:** ^1^ Sackler Institute of Pulmonary Pharmacology, Institute of Pharmaceutical Science, School of Cancer and Pharmaceutical Sciences, and; ^2^ MRC–Asthma UK Centre for Allergic Mechanisms in Asthma, Guy’s Hospital–King’s College London, London, United Kingdom; ^3^ Department of Pulmonary Physiology and Sleep Medicine, Sir Charles Gairdner Hospital, Nedlands, Western Australia, Australia; and; ^4^ Institute of Biomedical and Biomolecular Sciences, University of Portsmouth, Portsmouth, United Kingdom

**Keywords:** platelets, asthma, migration, allergen, CCR3

## Abstract

Platelet activation and pulmonary recruitment occur in patients with asthma and in animal models of allergic asthma, in which leukocyte infiltration, airway remodeling, and hyperresponsiveness are suppressed by experimental platelet depletion. These observations suggest the importance of platelets to various characteristics of allergic disease, but the mechanisms of platelet migration and location are not understood. The aim of this study was to assess the mechanism of platelet recruitment to extravascular compartments of lungs from patients with asthma and after allergen challenge in mice sensitized to house dust mite (HDM) extract (contains the DerP1 [*Dermatophagoides pteronyssinus* extract peptidase 1] allergen); in addition, we assessed the role of chemokines in this process. Lung sections were immunohistochemically stained for CD42b^+^ platelets. Intravital microscopy in allergic mice was used to visualize platelets tagged with an anti–mouse CD49b-PE (phycoerythrin) antibody. Platelet–endothelial interactions were measured in response to HDM (DerP1) exposure in the presence of antagonists to CCR3, CCR4, and CXCR4. Extravascular CD42b^+^ platelets were detected in the epithelium and submucosa in bronchial biopsy specimens taken from subjects with steroid-naive mild asthma. Platelets were significantly raised in the lung parenchyma from patients with fatal asthma compared with postmortem control-lung tissue. Furthermore, in DerP1-sensitized mice, subsequent HDM exposure induced endothelial rolling, endothelial adhesion, and recruitment of platelets into airway walls, compared with sham-sensitized mice, via a CCR3-dependent mechanism in the absence of aggregation or interactions with leukocytes. Localization of singular, nonaggregated platelets occurs in lungs of patients with asthma. In allergic mice, platelet recruitment occurs via recognized vascular adhesive and migratory events, independently of leukocytes via a CCR3-dependent mechanism.

**Clinical Relevance**

Platelets are activated in a distinct manner during asthma compared with hemostasis, leading to migration into inflamed lungs rather than to platelet-aggregatory events. Understanding mechanisms relating to platelet function might be exploited for novel therapies and will inform clinicians of the likely effectiveness of current antiplatelet drugs.

Platelets are traditionally considered as the main effector cells in hemostasis and thrombosis. However, there is accumulating evidence that platelets can participate and respond to immune and inflammatory signals (1), as well as contributing to repair and remodeling of tissue after injury (1).

Circulating activated platelets and platelet–leukocyte complexes have been reported in the blood of allergic subjects with asthma and in BAL fluid (2–6), and there is evidence of roles for platelets in other allergic or eosinophilic diseases: for example, in the immune response to parasitic infection, allergic rhinitis, and, recently, eosinophilic esophagitis (7, 8). We and others have demonstrated that platelets are required for leukocyte recruitment to the lungs (5, 9–13); contribute to mechanisms of allergen sensitization (12, 14, 15), bronchial hyperresponsiveness, and altered lung function (10, 13, 16); and induce features of airway remodeling such as extracellular-matrix deposition and increased airway smooth muscle (ASM) mass (17). Platelets are also a source of preformed mediators, such as cytokines, chemokines, adhesion proteins, and spasmogens, as well as growth factors, demonstrating that they have the capacity to participate in several physiological processes other than hemostasis after activation (18). We have previously reported that platelets derived from allergic donors undergo chemotaxis toward specific allergens *in vitro* and that pulmonary platelet accumulation and activation occurs directly in response to allergen exposure via IgE in a murine model of allergic airway inflammation (19). Furthermore, a population of platelets was considered not to be associated with leukocytes when examined histologically, suggesting that platelet migration could be a distinct event (19). Coordination of cellular migration in tissue undoubtedly requires navigation through tissue in a temporal–spatial manner with a prioritization of different chemotactic signals (20, 21). Despite the expression of CCR3, CCR4, and CXCR4 chemokine receptors on platelets (22), receptors that have also been reported to control myeloid-cell migration during allergic inflammation (23–26), the relevance of these chemokine receptors to platelet migration in the context of allergic inflammation remains unknown.

Given that platelets have been reported in extravascular compartments in allergic animal models (19) and have been found in the BAL fluid of patients with asthma (2), the aims of the present study were, first, to examine the compartmental location of platelets and quantify them in the airways of subjects with asthma and, second, to assess the active recruitment of platelets in a mouse model of allergic inflammation and the influence of platelet CCR3, CCR4, and CXCR4 chemokine receptors. In mice sensitized to the DerP1 (*Dermatophagoides pteronyssinus* extract peptidase 1) enzyme allergen from house dust mite (HDM) extract via the nasal route, platelet recruitment to the lungs was analyzed in fixed tissue, and the characteristics of platelet adhesion to the vasculature using a cremaster-muscle preparation with real-time intravital video microscopy were studied after local DerP1 challenge and after the administration of antagonists to CCR3, CCR4, and CXCR4 chemokine receptors. Parts of the data represented in this manuscript are in the King's College London institutional repository for the Ph.D. thesis of Dr Sajeel Shah (27).

## Methods

### Tissue Collection and Processing

The bronchial-biopsy component of the study was approved by the national research ethics committee at Guy’s and St. Thomas’ Hospitals (Research Ethics Committee: REC 08/H0804/67 and 10/H0807/99). Eight subjects without asthma (median [SD] age, 60 [22] y; 6 males/2 females); 12 subjects with steroid-naive, atopic, mild asthma (median [SD] age, 25 [5] y; 7 males/5 females; median [SD] forced expiratory volume in 1 s [FEV_1_] percent predicted, 87% [15%]), some of whom were from a previously reported study (28); and subjects with mild-to-moderate asthma (median [SD] age, 42 [12] y; 3 males/0 females; median [SD] FEV_1_% predicted, 76% [11%]) were recruited and underwent fiberoptic bronchoscopy. Tumor-free lung tissues were also taken from subjects undergoing lung resection for bronchial carcinoma (REC: 08/H0407/1), processed and sectioned by Dr. Amanda Tatler and Professor Alan Knox (University of Nottingham), and labeled as controls without asthma. All lung tissue was formalin fixed and paraffin-wax embedded before sectioning. Postmortem human lung tissue from patients without asthma, patients with nonfatal asthma, and patients with fatal asthma from the Airway Disease Biobank (processed and sectioned by A.L.J. and J.G.E.; Sir Charles Gairdner Hospital, Western Australia, Australia) was also used. The demographic characteristics of patients are shown in Table 1.

**Table 1.** Subject Demographics for Bronchial Biopsy and Tissue Collection

Tissue Code	Phenotype (Atopy Status)	Source	Age (Sex)	FEV_1_% Predicted	Respiratory Therapy
1	Mild asthma (A)	Biopsy	25 (M)	85	β_2_-Agonist
2	Mild asthma (A)	Biopsy	25 (F)	80	β_2_-Agonist
3	Mild asthma (A)	Biopsy	29 (M)	120	β_2_-Agonist
4	Mild asthma (A)	Biopsy	22 (F)	75	β_2_-Agonist
5	Mild asthma (A)	Biopsy	36 (M)	87	β_2_-Agonist
6	Mild asthma (A)	Biopsy	32 (F)	110	β_2_-Agonist
7	Mild asthma (A)	Biopsy	21 (F)	105	β_2_-Agonist
8	Mild asthma (A)	Biopsy	22 (M)	83	β_2_-Agonist
9	Mild asthma (A)	Biopsy	31 (M)	90	β_2_-Agonist
10	Mild asthma (A)	Biopsy	22 (M)	105	β_2_-Agonist
11	Mild asthma (A)	Biopsy	22 (M)	96	β_2_-Agonist
12	Mild asthma (A)	Biopsy	31 (F)	109	β_2_-Agonist
13	Moderate asthma (A)	Biopsy	50 (M)	67	β_2_-Agonist/steroid naive
14	Moderate asthma (A)	Biopsy	42 (M)	89	ND
15	Moderate asthma (A)	Biopsy	27 (M)	76	Combination therapy
16	Nonasthma (NA)	Biopsy	20 (M)	124	None
17	Nonasthma (A)	Biopsy	35 (M)	105	None
18	Nonasthma (A)	Biopsy	27 (F)	95	None
19	Nonasthma (ND)	Lung resection	57 (M)	ND	ND
20	Nonasthma (ND)	Lung resection	62 (M)	ND	ND
21	Nonasthma (ND)	Lung resection	75 (M)	ND	ND
22	Nonasthma (ND)	Lung resection	64 (F)	ND	ND
23	Nonasthma (ND)	Lung resection	77 (M)	ND	ND
24	Nonasthma (NA)	Postmortem	44 (M)	ND	ND
25	Nonasthma (NA)	Postmortem	26 (M)	ND	ND
26	Nonasthma (NA)	Postmortem	19 (F)	ND	ND
27	Nonasthma (NA)	Postmortem	34 (F)	ND	ND
28	Nonasthma (ND)	Postmortem	65 (M)	ND	ND
29	Nonasthma (NA)	Postmortem	71 (F)	ND	ND
30	Nonasthma (NA)	Postmortem	76 (M)	ND	ND
31	Nonasthma (A)	Postmortem	33 (F)	ND	ND
32	Nonfatal asthma (A)	Postmortem	27 (M)	ND	β_2_-Agonist
33	Nonfatal asthma (ND)	Postmortem	32 (F)	ND	β_2_-Agonist
34	Nonfatal asthma (NA)	Postmortem	34 (F)	ND	β_2_-Agonist
35	Nonfatal asthma (A)	Postmortem	21 (M)	ND	β_2_-Agonist
36	Nonfatal asthma (A)	Postmortem	18 (M)	ND	ND
37	Nonfatal asthma (NA)	Postmortem	29 (F)	ND	ND
38	Nonfatal asthma (A)	Postmortem	22 (M)	ND	β_2_-Agonist
39	Nonfatal asthma (ND)	Postmortem	78 (F)	ND	β_2_-Agonist, GC
40	Nonfatal asthma (NA)	Postmortem	15 (M)	ND	β_2_-Agonist
41	Nonfatal asthma (NA)	Postmortem	13 (F)	ND	β_2_-Agonist
42	Fatal asthma (A)	Postmortem	76 (M)	55	β_2_-Agonist, GC
43	Fatal asthma (A)	Postmortem	69 (M)	52	β_2_-Agonist, GC
44	Fatal asthma (A)	Postmortem	23 (F)	ND	β_2_-Agonist
45	Fatal asthma (A)	Postmortem	73 (F)	ND	β_2_-Agonist
46	Fatal asthma (A)	Postmortem	20 (F)	ND	β_2_-Agonist, GC
47	Fatal asthma (A)	Postmortem	34 (M)	81	β_2_-Agonist, GC
48	Fatal asthma (A)	Postmortem	33 (F)	ND	β_2_-Agonist, GC
49	Fatal asthma (ND)	Postmortem	18 (F)	ND	β_2_-Agonist, GC
50	Fatal asthma (ND)	Postmortem	10 (F)	ND	β_2_-Agonist, GC

*Definition of abbreviations*: A = atopic; F = female; FEV_1_ = forced expiratory volume in 1 second; GC = glucocorticosteroids (inhaled or oral); M = male; NA = nonatopic; ND = no data or data not collected.

“Combination therapy” was defined as use of a long-acting β_2_-agonist and inhaled corticosteroid.

### Murine Model of Allergic Airway Inflammation

Animal experiments were approved and performed under the Animals (Scientific Procedures) Act of 1986, amended UK regulations of 2012, and the Animal Welfare and Ethics Committee at King’s College London. Male BALB/c inbred mice (20–25 g) between 6 and 10 weeks of age were procured from Covance Laboratories. HDM extract containing the DerP1 allergen at 54 μg of DerP1 per 1 mg of total protein (Stallergenes Greer) was used for sensitization and challenge (29; *see* data supplement).

### Intravital Microscopy of the Cremaster Muscle

To achieve visualization of platelet interactions with the vasculature, a cremaster-muscle preparation was chosen as a stable method for real-time microscopy. Hamster anti–mouse CD49b-PE (phycoerythrin)–conjugated antibody (BD Biosciences), which has been used for intravascular platelet recording in previously published work (30), was injected intravenously (1.6 μg/0.1 ml) 1 hour before cremaster-muscle dissection. Details of the surgery used for intravital microscopy and video capture are provided online (*see* data supplement).

Sham-sensitized and HDM-sensitized mice were administered saline or HDM (100 μg/100 μl) subcutaneously (s.c.) to the scrotum on Day 13. The cremaster muscle was then dissected, and video-capture microscopy was conducted 24 hours after HDM administration before tissue was fixed with 3.7% paraformaldehyde (PFA) for processing and histological staining to detect leukocytes, eosinophils (31), and platelets (*see* data supplement).

Chemokine receptor antagonists were used in some *in vivo* experiments. SB328437, C-021, and AMD3100 are highly selective antagonists of CCR3, CCR4, and CXCR4 receptors, respectively (32–34). Doses of 30 mg/kg of SB328437, 30 mg/kg of C-021 and 10 mg/kg of AMD3100 were determined on the basis of previous literature in which these antagonists showed efficacy *in vivo* (33, 35–40).

### Data Analysis

Data are presented as the mean ± SEM as indicated, with data having been tested for normal distribution. Raw data values were compared using a Student’s *t* test or one-way ANOVA with a Bonferroni’s *post hoc* test as appropriate. (Prism; GraphPad Software). Probability values less than 0.05 were considered to indicate statistical significance.

## Results

### Increased Platelet Numbers Found in Airway Compartments of Subjects with Asthma

**Figure 1.**
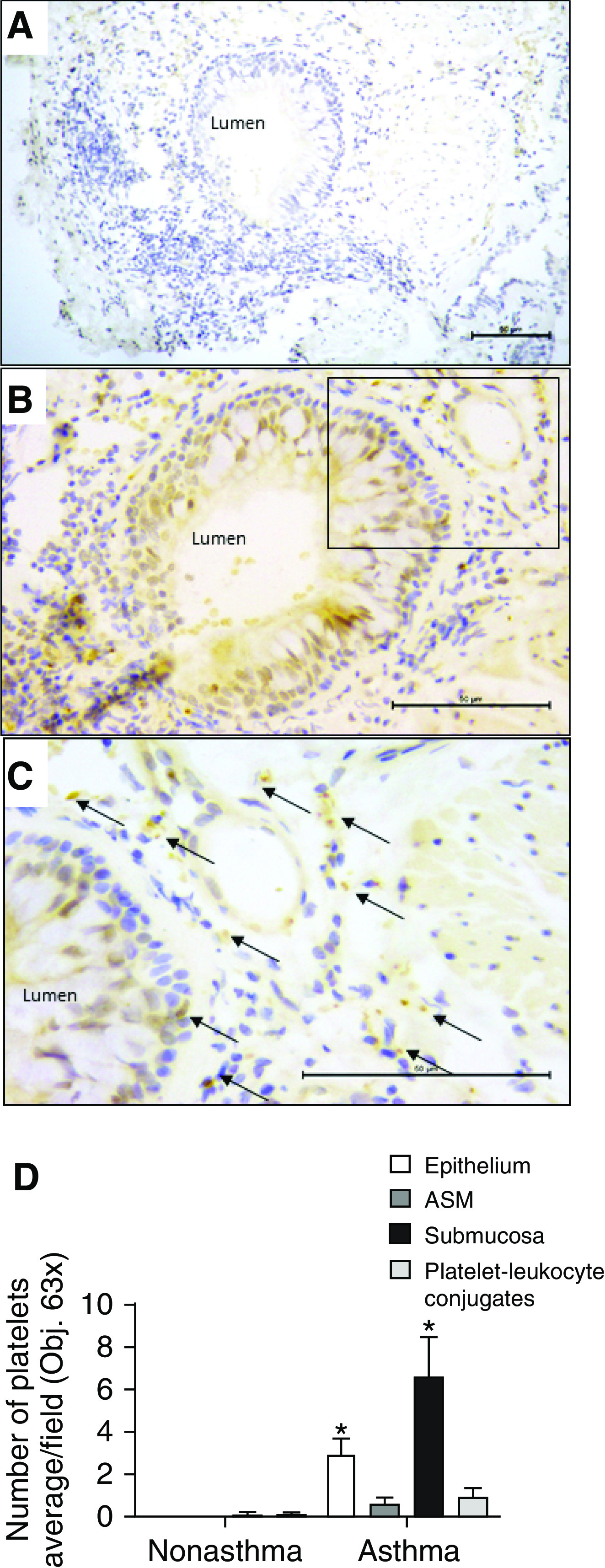
Platelet infiltrates in biopsy specimens of patients with asthma. Sections of lungs from patients with mild asthma (steroid-naive) or subjects undergoing lung resection for bronchial carcinoma were immunostained with the platelet-specific CD42b antigen. (*A*) Section of lung from a patient with mild asthma stained in the absence of primary antibody. Scale bar, 60 μm. (*B*) Section of lung revealing the presence of platelets. Scale bar, 60 μm. (*C*) Expanded image of tissue within the rectangle in *B*. Scale bar, 60 μm. Arrows indicate the localization of platelets (stained brown). (*D*) Quantification of average platelet numbers within specific airway compartments of tumor-free subjects without asthma (*n* = 5) and subjects with steroid-naive mild asthma (*n* = 9) within the epithelial layer, airway smooth muscle (ASM) bundles, and submucosa and incidence of platelet–leukocyte conjugates. Photomicrographs show scale bars and have been manipulated using a Microsoft PowerPoint tool to enhance their “contrast” and “sharpness.” Platelets were manually counted at 630× magnification from each whole-biopsy section. Data are presented as the mean ± SEM (*n* = 5–9). *P < 0.05. Obj. = objective lens.

Lung biopsy slices resected from patients with mild atopic asthma revealed the presence of extravascular CD42b^+^ platelets in the submucosa (*P* < 0.05; 6.6 ± 1.9 platelets per field of view; *n* = 9) and within the epithelial layer (*P* < 0.05; 3.0 ± 0.7 platelets per field of view; *n* = 9), which appeared mostly as singular events compared with their appearance in nonasthmatic tissue (Figures 1A–1D). This accumulation of platelets was greater than that detected in areas of ASM or in areas complexed to leukocytes (Figure 1D). Thus, the site-specific presence of platelets in the lung tissue of patients with asthma is in contrast to the absence of platelets found in any lung compartments of tumor-free tissue from subjects without asthma with lung cancer (Figure 1D).

**Figure 2.**
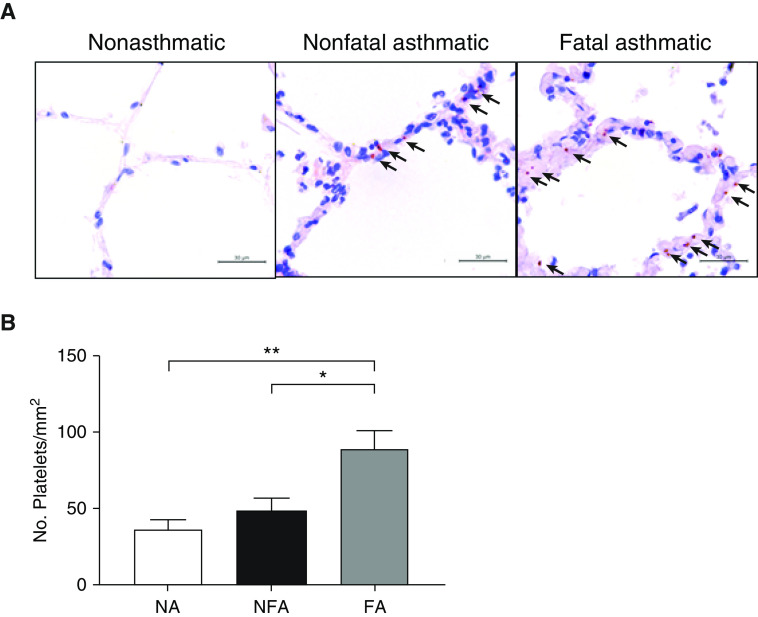
Immunostaining of CD42b^+^ platelets in human lung samples obtained postmortem. Sections of formalin-fixed lungs from patients with no asthma (NA), nonfatal asthma (NFA), and fatal asthma (FA) taken postmortem were stained with antihuman CD42b antibody to specifically detect platelets via immunohistochemistry. (*A*) Representative photomicrographs of lungs from patients with NA, NFA, and FA, with CD42b^+^ platelet events shown in magenta (highlighted with arrows). Scale bars, 30 μm. (*B*) The No. of platelet events in lung sections is expressed per millimeter squared (10 fields of view per section). Data are presented as the mean ± SEM (*n* = 8–10). *P < 0.05 and **P < 0.01. No. = number.

**Figure 3.**
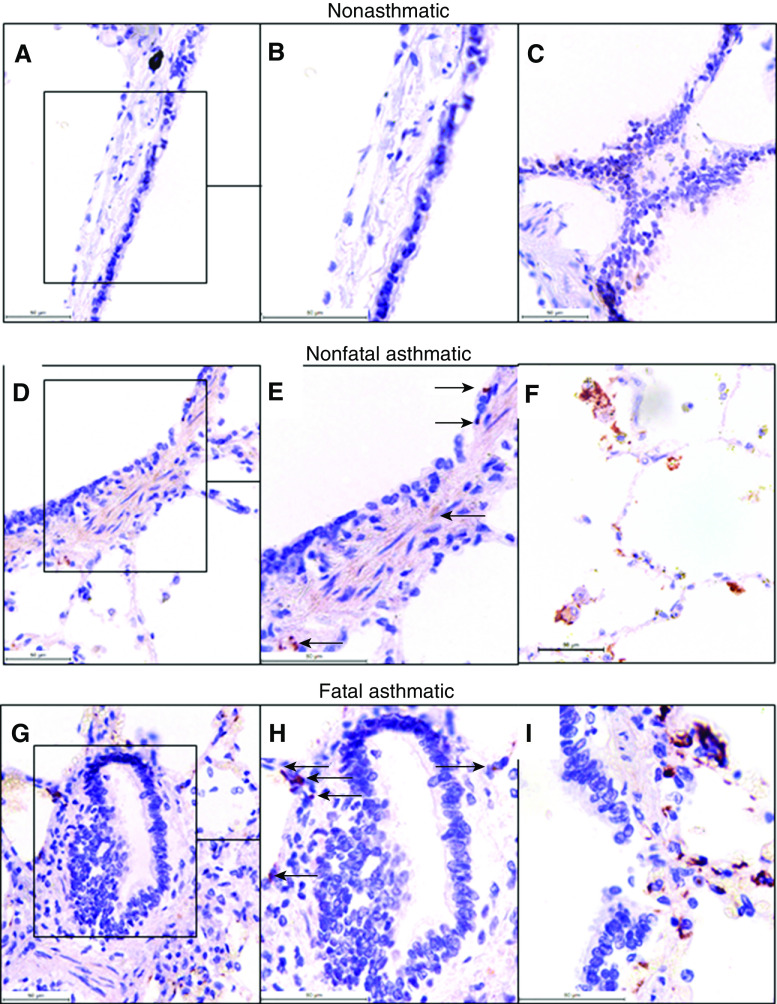
Representative immunohistochemistry of CD42b^+^ platelets localized to airway walls postmortem. Sections of human lungs from patients with NA, NFA, and FA obtained postmortem were stained with antihuman CD42b antibody to specifically detect platelets via immunohistochemistry. Images showing magenta CD42b^+^ platelet-staining events are displayed. (*A*–*C*) Representative images from NA lung sections: section of airway wall (*A*), magnified image of airway-wall section within the rectangle in *A* (*B*), and image of the parenchyma (*C*). (*D*–*F*) Representative images of sections from patients with NFA: section of airway wall (*D*); magnified image of airway-wall section within the rectangle in *D*, with arrows indicating platelets (*E*); and image of the parenchyma (*F*). (*G*–*I*) Representative images from FA lung sections: section of airway wall (*G*); magnified image of the airway-wall section in *G*, with arrows indicating platelets (*H*); and image of the parenchyma (*I*). All images have a representative scale bar, 50 μm. Sections *A*, *D*, and *G* are at 400× magnification, and images *B*, *E*, and *H* have been zoomed to 150% from originals. Sections *C*, *F*, and *I* are at 630× magnification.

A second distinct set of lung biopsy sections taken postmortem from patients without asthma (*n* = 8), patients with nonfatal asthma (*n* = 10), and patients with fatal asthma (*n* = 9) were also stained using immunohistochemistry to test for the presence of platelets. In sections taken from patients who died of asthma, platelets were observed in the alveolar walls and in the alveolar space as single, nonaggregated entities (Figure 2A). Platelet numbers in the lungs of patients with nonfatal asthma compared with patients without asthma were raised, although not significantly (no asthma vs. nonfatal asthma: 36.3 ± 6.4 platelets/mm^2^ vs. 48.8 ± 7.9 platelets/mm^2^) (Figure 2B). However, there was a significantly elevated number of platelets in tissues obtained from patients with fatal asthma compared with subjects without asthma (no asthma vs. fatal asthma: 36.3 ± 6.4 platelets/mm^2^ vs. 88.6 ± 12.2 platelets/mm^2^, *P* < 0.01). The presence of airway-wall platelets occurred with insufficient frequency in lung sections to determine the localization of platelets to specific airway compartments and perform statistical analyses. However, in concordance with studies of biopsy specimens from patients with mild, atopic asthma, platelets were distributed around the airway walls in histological sections from patients with nonfatal asthma and from patients who had died of asthma but were absent in sections from patients without asthma (Figure 3). Single platelets, and platelets associated with leukocytes, were also distributed within the parenchyma in patients with nonfatal asthma and in patients who had died of asthma (Figure 3). There was no incidence of platelet aggregates within the blood vessels of these patients.

### Studies in Allergic Mouse Lungs

**Figure 4.**
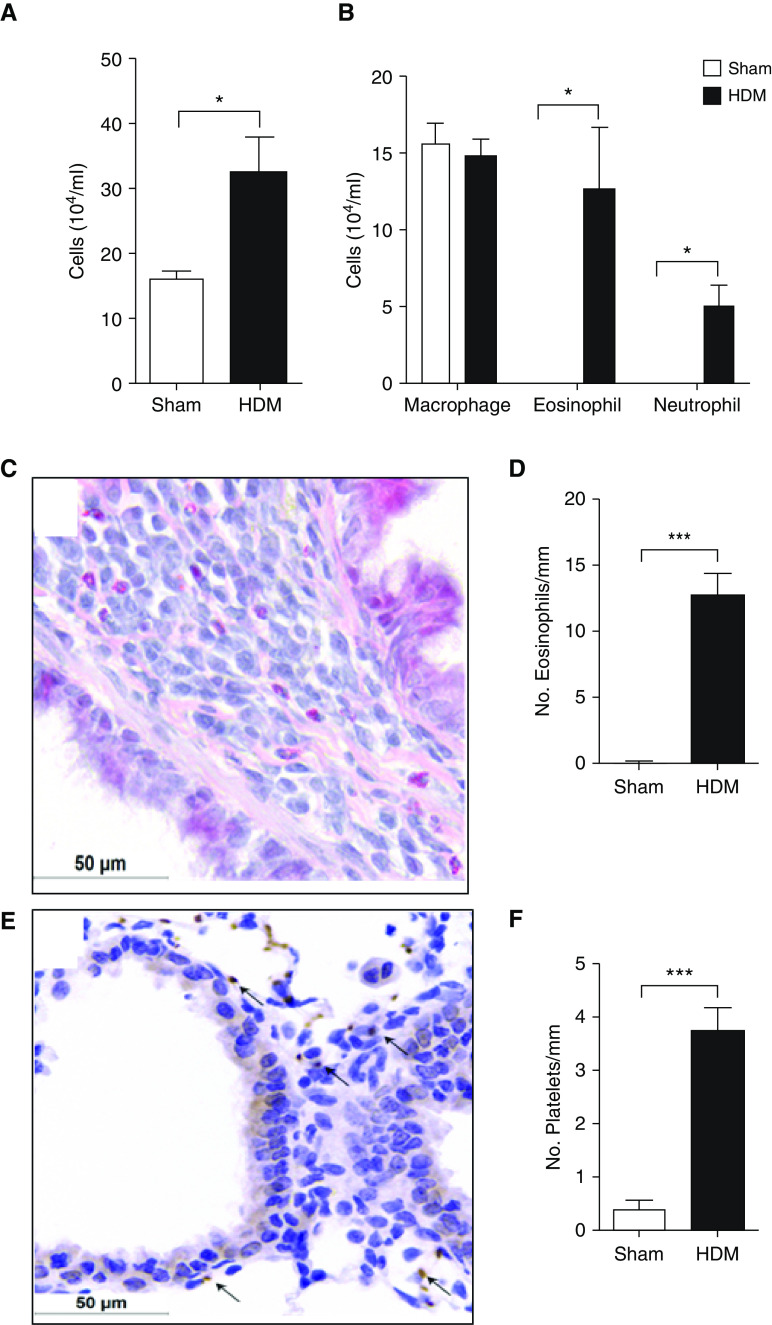
The effects of house dust mite (HDM) sensitization and exposure on leukocyte recruitment to the lungs. Sham- or HDM-sensitized (25 μg) mice were sensitized on Days 0, 1, 2, 3, 4, 7, 8, 9, 10, and 11 and were exposed again (challenged) with HDM (25 μg i.n.) on Day 13. BAL fluid was collected 24 hours after challenge. (*A*) Total BAL leukocytes. (*B*) Differential cell counts. Lungs were processed into sections for histological identification of eosinophils or CD42b^+^ platelets (immunohistochemistry). Representative images of (*C*) eosinophils stained red or (*E*) platelets stained brown (arrows) within the airway wall of HDM-sensitized and HDM-challenged mouse lungs. The No. of (*D*) eosinophils or (*F*) platelets located within the airway wall was then quantified and expressed per millimeter-length of airway wall. Data are presented as the mean ± SEM (*n* = 4). All images are at 630× magnification. Scale bars, 50 μm. *P < 0.05 and ***P < 0.001 compared with sham-sensitized mice.

To demonstrate the accumulation of platelets in the lungs in response to a clinically relevant allergen, mice were sensitized and exposed to DerP1 allergen from HDM extract. Pulmonary recruitment of leukocytes occurring in DerP1-sensitized mice compared with sham-sensitized mice was evident 24 hours after allergen challenge (Figure 4A). A significant proportion of these recruited leukocytes were eosinophils and neutrophils (sham vs. DerP1: 0 ± 0 × 10^4^ eosinophils/ml vs. 12.6 ± 8.0 × 10^4^ eosinophils/ml, *P* < 0.05; 0 ± 0 neutrophils/ml vs. 5.0 ± 2.7 neutrophils/ml, *P* < 0.05) (Figure 4B). Furthermore, use of Luna staining allowed eosinophils to be distinguished in histological sections (Figure 4C), where eosinophil numbers within the airway walls were raised in DerP1-sensitized and exposed mice (*P* < 0.001; Figure 4D). Lung sections immunohistochemically stained for CD42b^+^ platelets (Figure 4E) also revealed raised platelet numbers located within the airway walls of DerP1-sensitized and exposed mice (sham vs. DerP1: 0.4 ± 0.2 platelets/mm vs. 3.8 ± 0.4 platelets/mm, *P* < 0.001) (Figure 4F). These platelets appeared as singular entities, with no evidence of platelets forming aggregates.

### Studies to Observe Platelet Tissue Recruitment in Response to an Allergen Using Intravital Microscopy of the Murine Cremaster Muscle *In Vivo*

**Figure 5.**
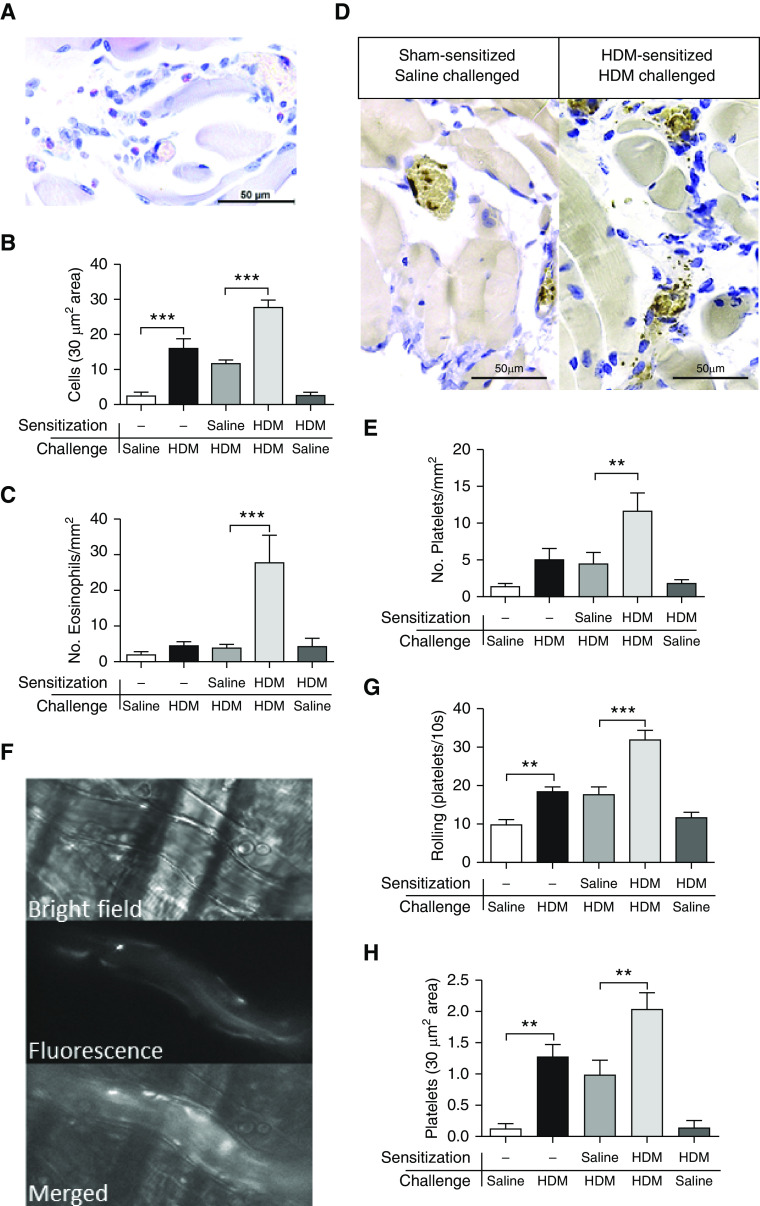
HDM sensitization and exposure induce rolling, adhesion, and migration of singular platelets. Sham, sham-sensitized, or HDM-sensitized (25 μg i.n.) mice were sensitized on Days 0, 1, 2, 3, 4, 7, 8, 9, 10, and 11 and were challenged with saline or HDM (100 μg) administered subcutaneously (s.c.) to the scrotum on Day 13. The cremaster muscle was then extracted and processed 24 hours later for histological evaluation of eosinophils (Luna staining) or CD42b^+^ platelets (immunohistochemistry). (*A*) Representative image of eosinophils stained red (scale bar, 50 μm) or (*D*) representative image of platelets stained brown within the cremaster muscle of HDM-sensitized and HDM-challenged mice (scale bars, 50 μm). Enumeration of (*B*) leukocytes, (*C*) eosinophils, and (*E*) platelets in extravascular compartments of the cremaster muscle, expressed per millimeter-squared area of tissue. In other groups of mice, anti–mouse CD49b-PE (phycoerythrin)–conjugated antibody was administered (i.v.), and the cremaster muscle was exposed for recording by intravital microscopy to monitor endothelial-adhesion events on postcapillary venules (for 10 s, minimum of three recordings). (*F*) Video frames revealing representative images of postcapillary venules viewed under bright-field light, under 580-nm fluorescent light, and as merged displays using a 63× Obj. to detect platelets noncomplexed to adherent leukocytes. The (*G*) No. of platelet-rolling events and the (*H*) No. of platelet-adhesion events were quantified. Data are presented as the mean ± SEM (*n* = 4–7). **P < 0.01 and ***P < 0.001.

To study the process of platelet recruitment and migration to allergic inflammatory sites, an allergic model of the mouse cremaster muscle was developed using intravital microscopy. This was necessary to provide tissue for stable recording of platelet-sized events because of the inherent instability of the lungs. Naive, sham-sensitized, and DerP1-sensitized mice (as above) were administered 100 μg of HDM extract containing DerP1 or saline s.c. to the scrotum. The cremaster muscle was exposed, and videos of postcapillary venules were taken for intravital analysis and histological morphometry after 24 hours. Leukocyte migration to the extravascular compartment of the cremaster muscle was used to gauge the severity of the allergic inflammatory response. A comparison between acute saline and DerP1 administration (administered s.c.) in sham-sensitized mice revealed significantly elevated numbers of extravascular leukocytes in the cremaster muscle of DerP1-challenged mice (*P* < 0.001; Figures 5A and 5B). DerP1 sensitization (intranasal) and challenge with DerP1 administered locally (administered s.c. to the scrotum) led to a further significant elevation in the number of leukocytes present in the extravascular compartment of the cremaster muscle (*P* < 0.001; Figure 5B). The increased inflammatory response in DerP1-sensitized mice indicated that allergic inflammation was prevalent. A DerP1-sensitized and saline-exposed group was included to act as a further control, showing comparable leukocyte recruitment to the acute-saline control group. Furthermore, eosinophil recruitment was significantly raised in DerP1-sensitized and DerP1-challenged mice, compared with sham-sensitized, DerP1-challenged mice (*P* < 0.001; Figure 5C). As the recruitment of eosinophils is a distinguishing feature in allergic inflammation, these data support the conclusion that DerP1 challenge in the cremaster muscle of DerP1-sensitized (via the intranasal route) mice is capable of causing an allergic inflammatory response. Cremaster tissue was also processed for immunohistochemical staining to detect platelets (Figure 5D). DerP1 sensitization led to significant platelet migration in the extravascular regions of the cremaster muscle compared with sham sensitization after DerP1 challenge (*P* < 0.01; Figure 5E). These data show that platelet accumulation occurred as a result of allergic inflammation in the cremaster muscle, which is likely preceded by distinct platelet-rolling and platelet-adhesion events.

Platelets were tagged with anti–mouse CD49b-PE–conjugated antibodies in intravital microscopy of the cremaster muscle to allow for comprehensive assessment of the tracking of rolling and adhesion events on the endothelium of individual platelets that could be dissociated from leukocyte-recruitment events, which was ascertained using bright-field images of the same vessel location (*see* Figure 5F and Videos E1 and E2 in the data supplement). After DerP1 administration to the scrotum of healthy mice, platelet-adhesion and platelet-rolling events were both significantly raised compared with events after saline administration (saline challenge vs. DerP1 challenge: 10.2 ± 1.0 rolling events/10-s interval vs. 18.8 ± 1.1 rolling events/10-s interval, *P* < 0.01 [Figure 5G]; 0.1 ± 0.1 cells with platelet adhesion/30 μm^2^ vs. 1.3 ± 0.2 cells with platelet adhesion/30 μm^2^, *P* < 0.01 [Figure 5H]), indicating that increased platelet activity occurred in response to acute inflammatory cues. In DerP1-sensitized mice (via the intranasal route) and local (scrotum) DerP1-challenged mice, platelet-adhesion and platelet-rolling events were further significantly elevated (*P* < 0.001, Figure 5G; *P* < 0.01, Figure 5H), specifically signifying that allergen exposure had caused increased intravascular platelet rolling and adhesion of single platelets that were not associated with leukocytes. Furthermore, neither intravascular platelet aggregation nor embolus formation were evident in these video recordings.

### Platelet Tissue Recruitment, in the Absence of Interactions with Leukocytes, in Response to an Allergen Was Dependent on CCR3

**Figure 6.**
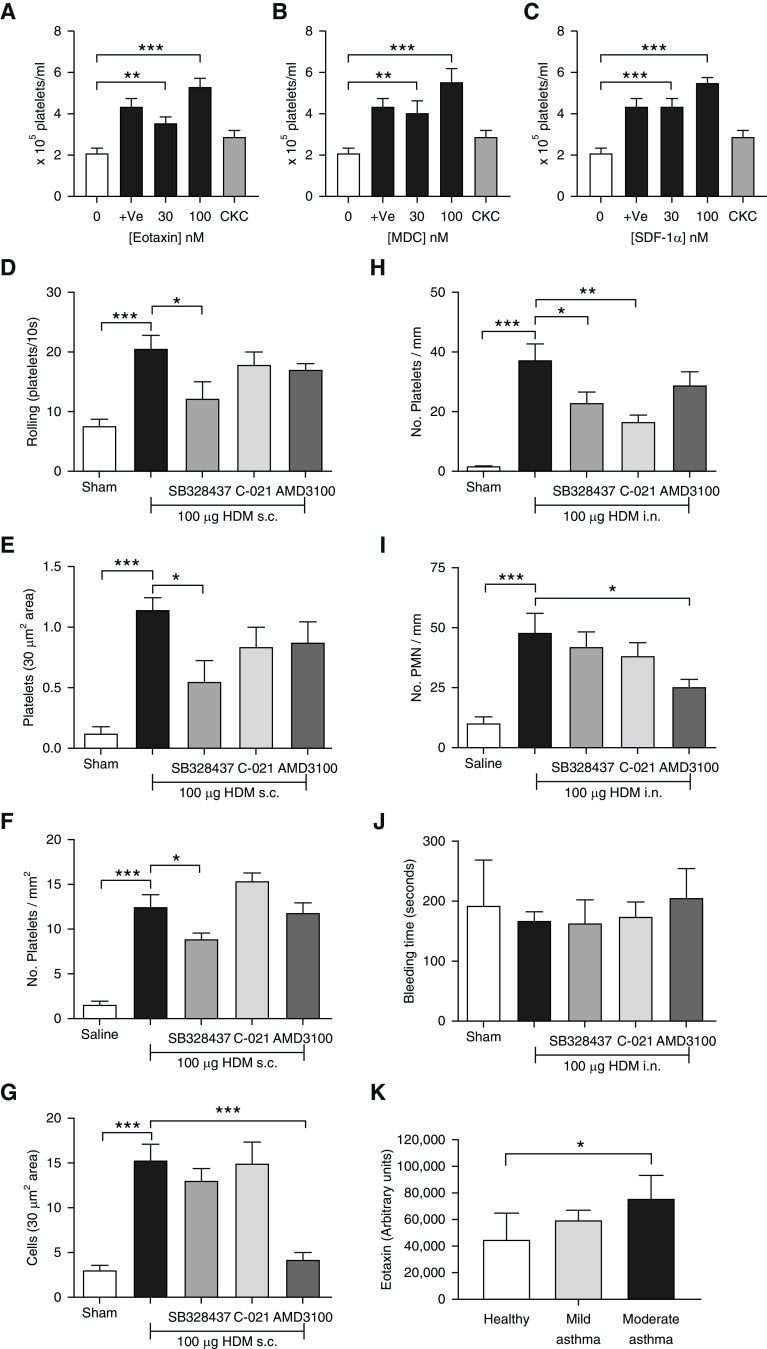
Platelet migration in response to allergen exposure is mediated by CCR3 and eotaxin. Washed platelets processed from citrated human blood were added to the top chamber of a Transwell-insert chemotaxis plate and were tested for their ability to migrate toward (*A*) eotaxin, (*B*) MDC, and (*C*) SDF-1α, which had been added to the bottom chambers. fMLP (plus vehicle [+Ve], 30 nM) acted as a positive control. Equimolar concentrations of fMLP (30 nM) added to both top and bottom wells served as a CKC. Platelet migration into the bottom chambers was counted after a 45-minute incubation. Responses to (*A*) eotaxin, (*B*) MDC, and (*C*) SDF-1α are represented as a chemotaxis index. In separate experiments, sham, sham-sensitized, and HDM-sensitized mice (25 μg of HDM i.n. on Days 0, 1, 2, 3, 4, 7, 8, 9, 10, and 11) were administered Ve, SB328437 (30 mg/kg i.p.), C-021 (30 mg/kg i.p.), or AMD3100 (10 mg/kg i.p.) on Day 13 at 30 minutes before saline or HDM (100 μg) challenge administered s.c. to the scrotum. On Day 14, anti–mouse CD49b-PE–conjugated antibody was administered (i.v.), and the cremaster muscle was exposed for recording by intravital microscopy to monitor endothelial-adhesion events on postcapillary venules (for 10 s, minimum of three recordings) to measure (*D*) platelet rolling and (*E*) platelet adhesion. The cremaster muscle was then dissected and processed to allow histological analysis of extravascular platelets using (*F*) a platelet-specific anti–mouse CD42b antibody or (*G*) staining for eosinophils (Luna stain). A further group of sham or HDM-sensitized mice (as above) were administered Ve, SB328437 (30 mg/kg i.p.), C-021 (30 mg/kg i.p.), or AMD3100 (10 mg/kg i.p.) on Day 20 at 30 minutes before saline or HDM (100 μg i.n.) challenge. On Day 21, the lungs were (*H*) dissected and processed to conduct immunohistochemistry to detect CD42b^+^ platelets and were (*I*) counter-stained with hematoxylin to enumerate PMN. (*J*) On Day 21, before culling, the tail was resected and immersed in warm saline to allow measurement of tail bleeding time. (*K*) Human explanted ASM bundles from healthy patients (*n* = 3), patients with mild asthma (*n* = 3), and patients with moderate asthma (*n* = 3) were grown in culture and treated with serum-free media; cell-free, cell-conditioned media was collected at 72 hours and applied to a cytokine-array membrane. Data show semiquantitative levels of eotaxin from duplicate dots on autoradiographs by densitometry. Data are expressed as the mean ± SEM (*n* = 6 in *A*–*C*, *n* = 6–8 in *D*–*G*, *n* = 8–12 in *H*–*J*, and *n* = 3 in *K*). *P < 0.05, **P < 0.01, and ***P < 0.001. CKC = chemokinetic control group; PMN = polymorphonuclear cells.

We have previously reported that platelet migration into the airways of allergen-sensitized mice occurs after allergen challenge via an FcεRI-IgE–dependent process, further reporting that platelets are able to undergo chemotaxis directly toward the sensitizing allergen (19). Platelets also express certain types of chemokine receptors (CCR3, CCR4, CXCR4) of pertinence to pulmonary cellular recruitment during asthma, although the physiological significance of these observations has not yet been fully elucidated (22). Platelets isolated from the blood of healthy human donors migrated in response to CCL11 (eotaxin) (*P* < 0.001; Figure 6A), CCL22 (MDC) (*P* < 0.001; Figure 6B), and CXCL12 (SDF-1α) (*P* < 0.001, Figure 6C), ligands for the CCR3, CCR4 and CXCR4 receptors, respectively. The magnitude of chemotactic activity was comparable with that in platelet responses toward the chemoattractant fMLP, as reported previously (41, 42; Figures 6A–6C), and the directionality of cell movement was tested through the addition of fMLP to the top and bottom well to induce a chemokinetic rather than chemotactic state (Figures 6A–6C). We next investigated the influence of these chemokine receptors on platelet adhesion and migration using intravital microscopy of the cremaster muscle of DerP1-sensitized mice after local HDM administration, with the mice having received prior administration of selective antagonists toward CCR3 (SB328437), CCR4 (C-021), or CXCR4 (AMD3100) receptors. SB328437 (30 mg/kg) significantly inhibited platelet rolling (*P* < 0.05; Figure 6D), platelet adhesion (*P* < 0.05; Figure 6E), and the extravascular presence of platelets (*P* < 0.05; Figure 6F). Neither administration of C-021 nor the administration of AMD3100 influenced allergen-induced platelet recruitment (Figures 6D–6F). However, antagonism of CXCR4 receptors did significantly inhibit leukocyte recruitment in this model (*P* < 0.0001; Figure 6G), revealing pathways of recruitment for platelets that were different from those for leukocytes. This difference in recruitment patterns was also apparent after intranasal administration of HDM in DerP1-sensitized mice, with the presence of extravascular platelets in the airways being significantly inhibited by SB328437 administration (*P* < 0.05; Figure 6H) as well as by C-021 administration (*P* < 0.01; Figure 6H); in contrast, leukocyte recruitment was inhibited with administration of AMD3100 only (*P* < 0.05; Figure 6I). Furthermore, neither the exposure to the allergen nor the inhibition of platelet recruitment through the administration of the CCR3 antagonist affected bleeding times in mice (Figure 6J), suggesting that platelet recruitment and activation were not results of changes in hemostasis, confirming findings of previous studies (43). Given the ability of eotaxin to induce platelet chemotaxis *in vitro* (Figure 6A), the role of CCR3 on platelet recruitment *in vivo*, and vascular adhesion and migratory events in the absence of interactions with leukocytes via intravital microscopy, we examined the ability of ASM derived from a phenotypically similar cohort of subjects with mild or moderate asthma (based on therapy, age, and FEV_1_% predicted) to express eotaxin. We show that eotaxin expression is significantly increased in cultured ASM derived from patients with moderate asthma compared with that derived from patients without asthma, and this appeared to be associated with the severity of asthmatic disease in the tissues biopsied and with which ASM bundles were dissected (*P* < 0.05; Figure 6K), as has been seen by others in the secretions of asthmatic airways (44–46).

## Discussion

This is the first report of the immunohistochemical analysis and quantification of platelets in human lung sections from patients with asthma and is the first visualization of real-time adhesion events of platelets to vessel walls in response to an allergen in a mouse model of allergic inflammation to show that platelet recruitment can occur in a manner that is similar to, yet distinct from, leukocyte recruitment. Heightened platelet recruitment to the lung parenchyma was evident in postmortem biopsy specimens from patients who had died of asthma, compared with tissue sections obtained from individuals with nonfatal asthma and individuals without asthma who died of other causes. These extravascular platelets appeared in abluminal regions of the alveolar wall and, in some cases, within alveolar air spaces, extending previous clinical and experimental reports that platelets can be found extravascularly in a variety of circumstances as single events not associated with leukocytes. Furthermore, ASM cells derived from biopsies of both mildly and moderately asthmatic lungs were shown to have increased secretion of eotaxin compared with control ASM cells, and given that this chemokine is a platelet chemoattractant with relevance to asthma and has been reported elsewhere in sputum and BAL fluid (44–46), this increase provides a link between patient-derived data and the mechanistic understanding of platelet recruitment into the lung from murine models. This recruitment of platelets was not associated with platelet aggregates either within blood vessels or extravascularly. Similarly, real-time video recordings of the cremaster circulation in response to an allergen in sensitized mice suggest that the platelet recruitment was not related to the classical platelet activation and aggregation associated with thrombosis and hemostasis and could occur independently of leukocyte adhesion. This finding is similar to our observation that platelet recruitment to the lungs after LPS administration can occur independently of neutrophil recruitment (47). Nevertheless, an association of platelets and neutrophils was observed extravascularly (as were lone platelets) (47). Thus, the dynamics of platelet recruitment and their subsequent extravascular association with other cell types are not fully understood, and this process may be different from the well-documented occurrence of intravascular platelet–leukocyte complexes during inflammation; and in patients with asthma, which is predictive of leukocyte (eosinophil) migration (5, 9). We cannot conclude on the basis of our current methodology that the intravascular association of platelets with leukocytes also leads to the extravasation of heterogeneous complexes.

An insufficient quantity of airway wall in our human lung sections meant we could not confidently determine whether there was platelet recruitment specifically to this lung compartment, although we did observe single, nonaggregated platelets in the airway walls of patients with nonfatal asthma and patients with fatal asthma. Clinically, platelets can be located within extravascular compartments of bronchial biopsy specimens, including on the epithelial surface (48) and in BAL fluid (2). However, this is the first study in which the ability to quantify the presence of platelets has been addressed through the use of platelet-specific immunohistochemistry while comparing different patient phenotypes, and our observations support other reports of extravascular platelets being observed in other inflammatory conditions (47, 49–52).

Circulating platelets have diminished stores of mediators as a result of increased activity toward inflammatory agonists in allergic asthma, which reflects the directed migration of platelets to the lungs of patients with asthma in the event of peripheral thrombocytopenia after allergen challenge (4, 53). Recent studies (10, 12) point to a primary role for platelet activation and degranulation in eosinophil recruitment. Therefore, it is interesting that there was a significantly increased number of platelets in parenchymal tissue in fatal, but not nonfatal, asthma. This parallels observations of eosinophil-staining events around membranous small airways, which determined that a significant increase in the number of eosinophils in patients with fatal asthma compared with healthy donors existed but that no obvious differences in lung sections between healthy individuals and patients with nonfatal asthma were apparent (54). Such studies are unable to measure cellular recruitment to the lungs in temporal relation to a relevant and specific stimulus and highlight the difficulty in measuring the incidence of inflammatory cells in lung samples taken from patients with nonfatal asthma who are prone to uncontrolled and undocumented exposure to (or the temporary absence of) inflammatory stimuli outside of the clinic. Thus, in contrast, with conditions of controlled exposure, platelet activation, and peripheral thrombocytopenia are induced in patients with asthma during allergen exposure with the HDM allergen DerP1, presumably as a result of localized pulmonary platelet recruitment (53). Future clinical protocols using controlled conditions over the complete time course of allergen exposure in patients with asthma would permit lung biopsy specimens to be fully elucidated with regard to the mechanisms and magnitude of platelet migration and would enable correlation of these with physiological parameters such as lung function and early- or late-phase responses, particularly as platelets are implicated in changes in lung function induced by allergen exposure (10, 13, 16), and with features of airway remodeling such as collagen deposition and increased smooth muscle mass (17). Although platelet numbers in the ASM layers of stable subjects with mild asthma were not found to be different from those of healthy control subjects, we believe that patients with more symptomatic asthma (patients with moderate-to-severe asthma or, indeed, patients with mild asthma after allergen exposure at a temporally relevant point) will have increased platelet numbers in their airways, as seen in our cohort with fatal asthma, particularly given that we and others have shown that patients with this phenotype of asthma showed significantly increased eotaxin levels in ASM compared with the healthy patients (44, 55, 56); this was further potentiated by extracellular-matrix proteins (57) and after inflammatory stimuli (57, 58), both of which are features of an exacerbated airway, confirming our findings in chronically challenged animal models of allergic inflammation (17, 19).

An *in vivo* allergen-sensitization and allergen-exposure model in mice was optimized to support and reaffirm the presence of extravascular platelets observed in human lungs. HDM is a clinically relevant source of an antigen (DerP1) necessary to develop *in vivo* models of allergen sensitization and inflammation via inhaled administration (29, 59). It was also noticeable that the singular administration of HDM extract induced an acute inflammatory response in naive mice. Reports have suggested that DerP1 also has proteolytic activity that might therefore propagate this acute, innate inflammatory response (60).

For the first time, an elevated number of extravascular platelets within the airway walls was measured after DerP1 sensitization and challenge in mice, which supports previous studies from our group showing an accumulation of platelets in the lung in ovalbumin-sensitized mice and chemotaxis toward allergens *in vitro* (19). It is now recognized that the migration of leukocytes is a highly orchestrated process, with different chemoattractive cues determining the spatial and temporal organization of migrating cells (20, 21). Whether the event of allergen sensitization affects platelet CCR3 activity (61) or the IgE-FcεRI interaction with platelet CCR3 occurs to further promote platelet migration is not known (62) but is worthy of investigation. Although it is recognized that CCR4 was also involved in platelet recruitment into the lungs, the absence of an effect on the cremaster muscle makes it difficult to understand whether CCR4 had a direct role on platelet adhesion in response to an allergic stimulus, and therefore makes it difficult to understand how platelet adhesion to the endothelium occurred. The recruitment of single, nonaggregated platelets to extravascular compartments of the lung in sensitized mice exposed to an allergen and the rolling and endothelial adhesion of platelets that were not complexed to leukocytes in response to local scrotal HDM challenge in the intravital cremaster preparation of HDM-sensitized mice suggest that platelets are recruited as a direct consequence of allergen exposure. Similarly, platelet events observed in lung tissue from patients who died of asthma were also single and nonaggregated. Although the atopic status of all the fatal asthma cases was not known, a preponderance (7 of 9) were cases of allergic asthma, and the presence of platelets in lung tissue therefore might have been the direct result of an allergen or inflammatory stimulus.

The cremaster muscle was chosen instead of the lungs as a stable platform to record platelet-adhesion events in response to an allergen, as lungs are inherently unstable because of their delicate structural nature, their motion due to respiratory maneuvers, their enclosed position within the body, and the inability to clearly visualize platelet-adhesion events (because of their small size) without the use of sophisticated technology and adaptions (47, 63) that have primarily focused on the pulmonary circulation of the lung parenchyma rather than on the bronchial circulation of airways (43, 64).The rationale for the use of an anti-CD49b antibody (rather than other platelet-specific antigens) to visualize platelets has been previously reported (30). Although it is acknowledged that other cell types might also express CD49b, the potential issue of background tissue positivity was not encountered here. Florescence levels of CD49b-PE appeared substantially high on platelets. Indeed, a low amount of bright-field light was necessarily applied before taking video recordings, allowing other blood components that presented with little to no fluorescence to be observed. The smaller, unique morphology of platelets also allowed them to be clearly distinguished from other blood cells.

To conclude, we have demonstrated the presence of single, extravascular platelets in the lung tissue of subjects with asthma and in the lungs of allergic mice after allergen challenge. At least in mice, this extravascular recruitment in the absence of interactions with leukocytes appears to be dependent on the activation of CCR3 receptors, implicating eotaxin in this process. Given that it is now appreciated that platelets are important for allergen sensitization, leukocyte recruitment, bronchospasm, and airway-wall remodeling in animal models of allergic inflammation, the presence of activated platelets in lung tissue may contribute to these activities and further confirms the likely importance of this cell type in this disease beyond the familiar requirement of platelets in hemostasis (65). Understanding the implications of this migration of platelets to the lungs as a result of allergic inflammation could therefore present novel drug targets to treat asthma because platelets become activated and directly contribute to lung pathophysiology via mechanisms unrelated to their well-recognized requirement during hemostasis (43, 66, 67).

## Supplementary Material

Supplements

Author disclosures
