# Supplementary material for: Platelets Independently Recruit into Asthmatic Lungs and Models of Allergic Inflammation via CCR3
Source: Am J Respir Cell Mol Biol. 2021 May;64(5):557–68. doi: 10.1165/rcmb.2020-0425OC (PMC8086046; doi:10.1165/rcmb.2020-0425OC)
Supplement: Supplements [file rcmb.2020-0425OC.html]

Platelets Independently Recruit into Asthmatic Lungs and Models of Allergic Inflammation via CCR3 | American Journal of Respiratory Cell and Molecular Biology

- disclosures.pdf (250 KB)
- shah\_data\_supplement.pdf (665 KB)
- shah\_et\_al.\_supplementary\_video\_1.avi (14 MB)
- shah\_et\_al.\_supplementary\_video\_2.avi (9 MB)
