# Supplementary material for: Platelets Independently Recruit into Asthmatic Lungs and Models of Allergic Inflammation via CCR3
Source: Am J Respir Cell Mol Biol. 2021 May;64(5):557–68. doi: 10.1165/rcmb.2020-0425OC (PMC8086046; doi:10.1165/rcmb.2020-0425OC)
Supplement: Supplements [file rcmb.2020-0425OC_shah_data_supplement.pdf]

ONLINE SUPPLEMENTARY DATA

Platelets Independently Recruit into Asthmatic Lungs and Models of Allergic Inflammation via CCR3

Sajeel A. Shah, PhD [1,y]; Varsha Kanabar, PhD [1]; Yanira Riffo-Vasquez, PhD [1]; Zainab Mohamed, BSc [1]; Simon J. Cleary, PhD [1,z]; Christopher Corrigan, MD [2]; Alan L. James, MD [3], John G. Elliot, MSc [3]; Janis K. Shute, PhD [4]; Clive P. Page, PhD [1]; Simon C. Pitchford, PhD [1].

[1]Sackler Institute of Pulmonary Pharmacology, King's College London, Institute of Pharmaceutical Science, School of Cancer and Pharmaceutical Sciences, 5th Floor, Franklin Wilkins Building, 150 Stamford Street, Waterloo Campus, London, SE1 9NH.

[2]MRC & Asthma UK Centre for Allergic Mechanisms in Asthma, King's College London, 5th Floor Tower Wing, Guy's Hospital, St. Thomas' Street, London SE1 9RT, UK.

[3] Department of Pulmonary Physiology and Sleep Medicine, Sir Charles Gairdner Hospital, Hospital Ave, Nedlands 6009, Western Australia

[4] Institute of Biomedical and Biomolecular Sciences, University of Portsmouth, White Swan Road, PO1 2DT, UK.

[y] Present address: Institute for Infection and Immunity, Office 2.231, St George's, University of London, 58 Cranmer Terrace, London, SW17 0QS, UK.

[z] Present address: University of California San Francisco (UCSF), Department of Medicine, San Francisco, USA

Platelet immunohistochemistry of human lungs.

Briefly, all lung tissue slides were passed through xylene, rehydrated and immersed in 3% hydrogen peroxide (H_2O_2) to block endogenous peroxidase activity and heated in sodium citrate buffer at 100°C for antigen retrieval. Slides were blocked with 1% BSA in PBS for 1 hour, and incubated with rabbit anti-human CD42b antibody (1:200, SP219 Abcam UK) in blocking buffer followed by incubation with biotinylated anti-rabbit IgG secondary antibody (1:200, Vector BA-1000) for 1hr. Control sections were incubated without primary antibody. Staining was visualized using either the Vectastain ABC HRP kit with DAB substrate (components A and B diluted 1:200) for another hour or developed using NovaRED Peroxidase (HRP) Substrate (Vector, UK) solution for 20 minutes, followed by a tap water wash. Sections were counterstained with Gill's haematoxylin and differentiated through immersion in acid alcohol solution and finally dehydrated and cleared in xylenes, before coverslipping with DPX mountant.

Eotaxin (CCL11) detection from human-cultured bronchial smooth muscle explants.

A chemokine array (RayBio-tech, Norcross, GA, USA) was employed to examine the levels of eotaxin produced by airway smooth muscle derived from asthmatic (mild and moderate) and non-asthmatic donors. The methods have been previously described (1). Briefly, cell-free cell-conditioned media from airway smooth muscle treated with serum-free media over 72hr was collected and added to the array membranes according to manufacturer instructions. Semi-quantitative levels of eotaxin from duplicate dots on autoradiographs (Amersham-Pharmacia, Amersham UK) that were developed from chemiluminescence exposure were quantified by densitometry and analysed using dot blot analysis program in ImageJ whereby the background is subtracted from the dot blots and the integrated density collected.

Human platelet chemotaxis assay.

Platelets were isolated from human peripheral venous blood obtained from healthy volunteers who had not taken NSAIDs or other anti-inflammatory drugs in the previous seven days, and were not prescribed anti-platelet drugs as we previously described (2). For all studies blood was collected in accordance with local ethical approval from King's College London and adhered to regulations outlined by the Human Tissue Act 2004. Platelets were prepared for the chemotaxis assay, using a 24-well transwell setup using inserts with membranes consisting of 3µm pores (Becton Dickinson) as previously described in detail (2). Platelet migration towards eotaxin, monocyte derived chemokine (MDC), and stromal cell derived factor-1α (SDF-1α) added to the bottom wells, was assessed at 30nM and 100nM after a 45 minute incubation. The chemo-attractant N-Formyl-methionyl-leucyl-phenylalanine (fMLP, 30nM) was added to the assay as a positive control in the bottom well (2,3), and as evidence for reversal of chemotaxis (chemokinesis CXC) in the bottom and top wells.

Sensitization of mice to HDM extract, processing and analysis of bronchoalveolar lavage (BAL) fluid.

Mice were anaesthetised with isoflurane before sensitization with HDM extract (25 µg per mouse, i.n.) or saline (25µL i.n.) on days 0, 1, 2, 3, 4, 7, 8, 9, 10 and 11 as described elsewhere (4). On day 13, mice were challenged with HDM extract (25µg per mouse, i.n.). 24 hours later mice were terminally anaesthetised with urethane (2.1g/kg i.p.) and the trachea cannulated for collection of BAL fluid. Total leukocyte numbers and cytospins for differential cells counts were performed. BAL fluid was mixed 1:1 with Türk's solution and the total leukocyte cell number quantified using a Neubauer improved haemocytometer under a phase contrast light microscope (Ziess Axiovert) with a 20x objective lens.

For differential cell quantification, BAL fluid was centrifuged using a Shandon Cytospin 3 on Superfrost Plus slides. The slides were then stained using Kwik Diff differential stain. The percentage of neutrophils, macrophages, lymphocytes and eosinophils was calculated by counting 200 cells from four random fields of view, using a phase contrast light microscope (Ziess Axiovert) with a 40x objective lens. The total number of each cell type was then calculated from the total cell counts.

In vivo administration of chemokine receptor antagonists.

The CCR3 receptor antagonist SB328437 (N-(1-Naphthalenylcarbonyl)-4-nitro-L-phenylalanine methyl ester) and the CCR4 receptor antagonist C-021 (2-[1,4'-Bipiperidin]-1'-yl-N-cycloheptyl-6,7-dimethoxy-4-quinazolinamine dihydrochloride) were both prepared in DMSO, while the CXCR4 receptor antagonist AMD3100 (1,1′-[1,4-Phenylenebis(methylene)]bis-1,4,8,11-tetraazacyclotetradecane octahydrochloride) was prepared in saline. Chemokine receptor antagonists were administered i.p. 30 minutes before allergen challenge, with control groups and the AMD3100 group, receiving equivalent amounts of DMSO. An allergen challenge with HDM (100 µg/100 µl) was administered s.c. to the scrotum in intravital microscopy preparations, and an allergen challenge with HDM (100 µg/100 µl) was administered i.n. in allergic lung studies.

Surgery for Intravital microscopy and video capture.

Mice were terminally anaesthetised with urethane and had their temperature regulated with a heated mat whilst surgery and video capture occurred. A small incision on the right ventral scrotal sac allowed extrusion of a single testis from the scrotal sac. The inferior part of the cremaster muscle surrounding the testis was pinned to the viewing window and an incision was made along the medial side of the cremaster muscle from the inferior pinned section. The cremaster muscle was then folded over laterally and pinned to the viewing window.

The cremaster muscle was mounted under a Zeiss Axioskop 2 inverted reflective fluorescence microscope with a digital CMOS ORCA-Flash 2.8 camera (Hamamatsu) and a water immersed 63x objective lens. The scale and area of cremaster muscle viewed was determined using a micro-ruler. The exposed tissue was superfused with 37°C Tyrode's buffer using a Watson-Marlow pump at 12 ml/min and excess Tyrode's buffer suctioned from the viewing window. Under 580 nm fluorescent light and a dimmed bright field light, both the platelets and the architecture of the post capillary venules could be clearly observed. Post capillary venules were distinguished from arterioles by

following the flow of blood to a convergence point, indicating the vessels under view were part of the venous blood circulation.

A minimum of 3 different post capillary venules from each mouse were recorded for 10 seconds. Platelet adhesion events were classified as instances where a fluorescent platelet remains bound to a section of endothelium for the duration of a 10 second video and expressed per area of cremaster muscle. Platelet rolling events were classified as instances where a fluorescent platelet slows down, stops or moves off the endothelium during the 10 second video.

Preparation and immunohistochemistry of mouse lungs.

The tracheas of anaesthetised mice were cannulated and lungs inflated with 0.5 ml of 3.7% paraformaldehyde (PFA). The lungs were dissected and stored in 3.7% PFA for 24 hours, before dehydration and paraffin wax embedding. 5 µm sections were cut using a Leica RM2125 RT rotary microtome and mounted on histological Superfrost Plus slides.

Staining for the platelet CD42b antigen was performed using the same method as for post mortem human lung tissue sections. However, anti-human CD42b antibody, that cross-reacts with mouse antigen, was diluted 1:500 and the NovaRED Peroxidase (HRP) step replaced. Sections were instead developed in DAB Peroxidase (HRP) Substrate Kit containing 0.3% H_2O_2 for 10 minutes, washed in tap water and the remainder of the protocol followed.

Sections of mouse lung were histologically stained with Luna stain to detect eosinophils, as described elsewhere (5). Tissue sections were passed through xylene and rehydrated. Sections were then immersed in Weigart's iron haemotoxylin kit solution for 2.5 minutes, washed with distilled water and differentiated in acid alcohol solution for 8 seconds. Sections were then washed with tap water and immersed in 0.5% Li_2CO_3, before dehydration steps and coverslip mounted with DPX mountant. The number of leukocytes present in extravascular compartments of cremaster muscle were counted and expressed per area of cremaster muscle.

Enumeration and localization of platelets in tissue sections.

For CD42b+ platelet enumeration, researchers were blinded to samples and platelets were manually counted at 63x magnification (objective) using a DM 2000 LED bright field

microscope with DFC295 camera (Leica). With bronchial biopsies, platelets were counted from each whole biopsy section per field of view. Platelets in non-asthmatic, non-fatal asthmatic and fatal-asthmatic patients lung sections, were enumerated from 10 fields of view per section and expressed per mm^2 of lung tissue (section) using ImageJ 1.48 (National Institutes of Health) analysis software to measure area.

In sham-sensitized and HDM-sensitized mice the number of extravascular CD42b+ platelet staining events between the basal lamina and the submucosal layer, was enumerated and expressed per mm along the airway wall using ImageJ 1.48 (National Institutes of Health) analysis software to measure length.

References

1. Kanabar V, Page CP, Simcock DE, Karner C, Mahn K, O'Connor BJ, Hirst SJ. Heparin and Structurally Related Polymers Attenuate eotaxin-1 (CCL11) Release From Human Airway Smooth Muscle. Br J Pharmacol. 2008; 154:833-42.

2. Amison RT, Jamshidi S, Rahman KM, Page CP, Pitchford SC. Diverse signalling of the platelet $P2Y_1$ receptor leads to a dichotomy in platelet function. Eur J Pharmacol. 2018; 827:58-70.

3. Czapiga M, Gao JL, Kirk A, Lekstrom-Himes J. Human platelets exhibit chemotaxis using functional N-formyl peptide receptors. Exp. Hematol. 2005; 33: 73–84.

4. Gregory LG, Causton B, Murdoch JR, Mathie SA, O'Donnell V, Thomas CP, Priest FM, Quint DJ, Lloyd CM. Inhaled house dust mite induces pulmonary T helper 2 cytokine production. *Clin Exp Allergy* 2009; 39: 1597-1610.

5. Luna LG. Histological staining methods for the differentiation of fungi. Med Lab (Stuttg). 1963; 16: 123-135.

Supplementary video 1: Representative video of intravascular platelet dynamics in the cremaster muscle of a mouse, sham-sensitized (25 µl saline i.n. on days 0, 1, 2, 3, 4, 7, 8, 9, 10, and 11) and challenged with saline s.c. to the scrotum on day 13. On day 14 anti-mouse CD49b PE-conjugated antibody was administered (i.v.), the cremaster muscle dissected and a post capillary venule recorded for 10 seconds, using a 63x objective lens. Video playback at 8 frames per second.

Supplementary video 2: Representative video of intravascular platelet dynamics in the cremaster muscle of a mouse DerP1-sensitized (25 µg HDM on days 0, 1, 2, 3, 4, 7, 8, 9, 10, and 11) and then HDM-challenged (100 µg s.c.) to the scrotum on day 13. On day 14 anti-mouse CD49b PE-conjugated antibody was administered (i.v.), the cremaster muscle dissected and a post capillary venule recorded for 10 seconds, using a 63x objective lens. Video playback at 8 frames per second.
